# Rare pediatric multi-system thrombosis post-COVID-19: a three-year follow-up case report and narrative review on rivaroxaban for long-term management

**DOI:** 10.1186/s13023-026-04382-7

**Published:** 2026-05-09

**Authors:** Shiyi Zhu, Xiaozhong Li, Ruyue Chen, Shuang Wu, Lu Jiang, Mengxia Li, Luyao Huang, Ningxun Cui

**Affiliations:** Department of Nephrology and Immunology, Children′s Hospital of Soochow University, Soochow, 215000 China

**Keywords:** Venous thromboembolism, COVID-19, Rivaroxaban, Pediatric anticoagulation, Thrombosis

## Abstract

This retrospective study presents a 10-year-old male with multi-systemic venous thromboembolism (VTE) secondary to COVID-19, including right ventricular thrombus(40 mm × 18 mm), bilateral iliac vein thrombosis, pulmonary embolism, and renal vein thrombosis. The child presented with fever, abdominal pain, and elevated inflammatory markers (CRP 222.72 mg/L, WBC 22.41 × 10^⁹^/L). Imaging confirmed extensive thrombi in the right ventricle, pulmonary arteries, and lower extremities. Anticoagulation with rivaroxaban (10 mg QD) was initiated alongside anti-infective and anti-inflammatory therapies. Over a 3-year follow-up, thrombus burden significantly regressed, with D-dimer decreasing from 73 430 µg/L to 290 µg/L, and no recurrence or bleeding complications were observed. Coagulation parameters (PT, APTT) stabilized within therapeutic ranges. This case suggests the efficacy and safety of rivaroxaban in long-term management of multi-system VTE in pediatric patient, especially in cases of COVID-19-associated hypercoagulability. The primary clinical outcomes were the resolution of thrombus burden as evidenced by imaging and the absence of bleeding complications or recurrence during the follow-up period. The findings align with emerging evidence supporting direct oral anticoagulants (DOACs) in children, emphasizing individualized dosing and long-term management. Further studies are warranted to validate DOACs’ role in pediatric thrombosis, especially in the context of viral infections.

## Clinical information

A 10-year-old male of Han Chinese ethnicity presented to the hospital with “fever with abdominal pain in the right shoulder and periumbilical region for 4 days”. The fever peaked at 39.6 °C, and blood tests at the hospital showed CRP 222.72 mg/L (0–8 mg/L), WBC 22.41 × 10^9^/L (4.6–11.9 × 10^9^/L), N 0.70 (0.32–0.71), and cardiology ultrasound showed solid right ventricular cavity (about 40 mm × 18 mm, oscillating with the cardiac cycle). He had recurrent fever for about 10–12 h with paroxysmal right shoulder muscle ache and abdominal pain around the umbilicus, malaise, poor appetite, paroxysmal cough.

His basic vital signs showed temperature 36.3℃, pulse 123 beats/min, respiratory rate 25 beats/min, blood pressure 127/55 mmHg, weight 47 kg, height 155.0 cm, BMI 19.6 kg/m², and pulse blood oxygen saturation 95%.

The child was pale, couldn’t walk for long and presented rhythmic grade II/6 systolic murmur audible between 2 and 3 ribs at the left sternal margin. He claimed periumbilical pain while without any palpable mass. The child was G1P1, full-term and normal delivery, with no abnormal birth history, no history of cardiovascular disease, thrombosis, or underlying disease. He had history of coronavirus infection, recurrent respiratory infections. The child has received 3 doses of the Sinopharm CNBG COVID-19 Inactivated Vaccine (Vero Cell) vaccine. No adverse reactions occurred following vaccination. Both of his parents are in good health and have no history of past illnesses.

Laboratory investigations on admission revealed a severe hyperinflammatory and hypercoagulable state. In addition to elevated CRP (190.6 mg/L) and WBC (21.02 × 10.

^9^/L), specific cytokine profiling indicated a cytokine storm phenotype: Interleukin-8 (IL-8) was elevated at 86.8 pg/mL (reference: 0–47 pg/mL), and Interleukin-1 Receptor Antagonist (IL-1RA) was 2683 pg/mL (reference: 0–2171 pg/mL). SAA was significantly elevated (> 240 mg/L). Coagulation assays showed a marked elevation in D-dimer (73,430 µg/L) accompanied by hypofibrinogenemia (Fibrinogen 1.52 g/L; reference: 1.8–3.5 g/L), suggesting consumption coagulopathy. **(**Table 1**)**

**Table 1:** Abnormal indicators on the first, seventh, ninth, twenty-first and thirty-seventh days after admission

Days	Blood Routine Examination	Blood Coagulation Indicators	Other Indicators	
Abnormal Indicators on Day 1 of Admission	CRP 190.6 mg/L	PT 16.6 s	SAA > 240.0 mg/L	lymphocyte subsets CD3 + CD25+ 4.59%
	WBC 21.02 × 10^9^/L	APTT 38.1 s	Hs-cTn 26.45 pg/mL	CD3-HLA-DR + 26.32%
	PLT 206 × 10^9^/L	INR 1.48	MYO 77.4 ng/mL	TCR + 10.21%
	N 0.79	AT-III 94.4%	KET 10 mm/L(++)	Antinuclear Antibody (titer) Positive
	LY 0.13	FIB 1.522 g/L	C3 2.08 g/L (0.85 − 0.1.93 g/L)	Antinuclear Antibody Granulotype 1:320
	PCT 0.23%	D-dimer 73 430 µg/L	C4 0.3 g/L (0.12–0.36 g/L)	ANCA Negative
	MPV 10.1 fL	FDP 272 100 µg/L	Occult Blood 50/L(++)	Anti-vascular Endothelial Cell Antibodies Negative
	PDW 13.5%	TAT 48.9 ng/ml	ALT 44.1 U/L	Mycoplasma pneumoniae IgM, IgG Negative
	Blood pH 7.196	PIC 23.98 µg/ml	TBIL 19.1 µmol/L	Antiphospholipid Heart Antibody Negative
		Fibrinogen Level 50.3 deg	DBIL 8.6µmol/L	Quantitatively Anti-beta2 Glycoprotein 1 Antibody Negative
		Composite Coagulation Index − 4.2	Blood Sedimentation 8 mm/h	HLA-B51 negative
		Platelet Aggregation Function: Normal	ASO Negative	Coronavirus Test Positive (Day 2)
			IL-1 RA 2683 pg/ml (0-2171 pg/ml)	Weight 47 kg / Height 155.0 cm / BMI 19.6 kg/m²
			IL-8 86.8 pg/ml (0–47 pg/ml)	
Abnormal Indicators on Day 7 of Admission	CRP 166.43 mg/L	PT 17.1 s	Procalcitonin 0.18 ng/ml	Coronavirus Test Positive
	WBC 10.06 × 10^9^/L	APTT 41.6 s	Lupus Anticoagulant Screening 68.5s	Blood Sedimentation 83 mm/h
	NEUT% 71%	INR 1.53	Lupus Anticoagulant Screening Confirmation Time 45.8s	Weight 47 kg / Height 155.0 cm / BMI 19.6 kg/m²
	Hb 84 g/L	FIB 4.311 g/L		
	PLT 292 × 10^9^/L	D-dimer 10 960 µg/L		
		TAT 11.8 ng/ml		
Abnormal Indicators on Day 9 of Admission	CRP 61.49 mg/L	PT 20.1s(PH)	IgA 3.26 g/L	Coronavirus Test Positive
	WBC 15.62 × 10^9^/L	APTT 37 s	IgG 20.61 g/L	Blood Sedimentation 10 mm/h
	Hb 82 g/L	FIB 0.159(PL)	Blood Potassium 3.3 mmol/L	Weight 47 kg / Height 155.0 cm / BMI 19.6 kg/m²
	PLT 174 × 10^9^/L	INR 1.82	Lactic Acid 2.7 mmol/L	
	NEUT% 76.2%	FIB 0.159 g/L	Alanine Aminotransferase 34.2 U/L	
	LY% 16.6%	D-dimer 54 990 µg/L	Aspartate Aminotransferase 51.3 U/L	
	Blood pH 7.572	FDP 207 610 ug/L	Lactate Dehydrogenase 605.3 U/L	
		TAT > 120 ng/mL	Creatine Kinase 721.2 U/L	
		PIC 18.53 ug/mL	Ferritin 717 pmol/L	
Abnormal Indicators on Day 20 of Admission	CRP1.2 mg/L	PT 80.8 s	Blood Potassium 3.3 mmol/L	Weight 47 kg / Height 155.0 cm / BMI 19.6 kg/m²
	WBC 8.58 × 10^9^/L	APTT 162.3 s	Blood Calcium 1.1 mmol/L	
	NEUT% 49.3%	INR 10.88	Lactic Acid 3 mmol/L	
	LY% 41.4%	FIB 0.27 g/L		
	Hb 90 g/L	TT 49 s		
	PLT 140 × 10^9^/L	AT-III 127%		
		D-dimer 14 850 µg/L		
		FDP 45 060 ug/L		
		TM 13.7 TU/mL		
		TAT 58.2 ng/mL		
		PIC 7.84 ug/ml		
Abnormal Indicators on Day 37 of Admission	CRP < 0.5 mg/L	PT 30.4 s	Weight 47 kg / Height 155.0 cm / BMI 19.6 kg/m²	
	WBC 4.79 × 10^9^/L	APTT 67.6 s		
	NEUT% 18.4%	INR 3.09		
	LY% 66%	D-dimer 1300 µg/L		
	ANC 0.88 × 10^9^/L	FIB 1.24 g/L		
	Hb 105 g/L	AT-III 127%		
	PLT 291 × 10^9^/L	TT 18.2 s		

The child’s genetic testing and whole exome high throughput sequencing had no specific genetic abnormalities. His pharmacogenetic testing recommended dose reduction for warfarin VKORC1, CYP4F2. Rivaroxaban ABCB1 could be routinely administered. His genetic testing (DNA) for pathogenic microorganisms was negative.

**Fig. 1:**
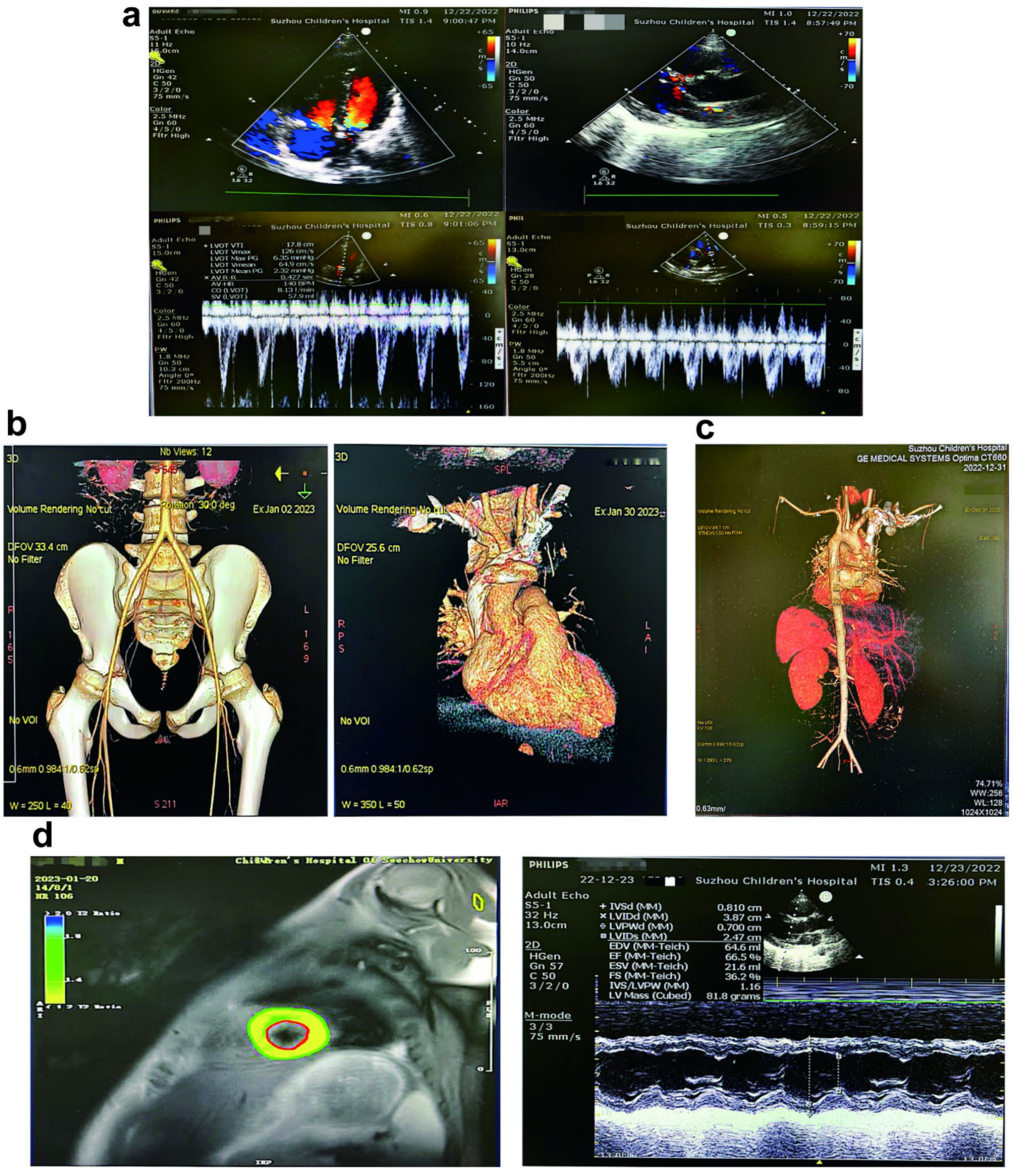
Selected imaging of the child’s initial admission to the hospital. (**a**) Cardiac ultrasound showed a 40 mm × 18 mm medium-high echogenic mass in the right ventricular cavity, with clear boundaries and irregular morphology, oscillating with the heartbeat, and a right ventricular free wall thickness of 3.9 mm. Pulmonary artery pressure was elevated, with mild tricuspid regurgitation and a pressure difference of 33mmHg. LVOT VTI: 21.1 cm, SV:55.1 ml, CO:6.62 L/min. (**b**) CT of the lower limbs showed marked thickening of the common iliac vein to the bilateral femoral veins with uneven density. PW measurement showed blood flow velocity in the common femoral vein Vmax = 0.09 m/s. (**c**) (**d**) CTA showed multiple emboli in both pulmonary arteries, posterior superior vena cava and right ventricular filling defects, and large, clumpy hyperdense shadows in the lower lobes of both lungs. (**d**) There were effusions in the bilateral thoracic and pericardial cavities

Chest radiographs showed a blurred deepening of the texture of both lungs, and a large lamellar high-density shadow in the right lung. Chest CT showed multiple lamellar hyperdense shadows in both lungs, bronchial insufflation sign, and inflammation in the lower lobes of both lungs, which was obvious in the right lower lung. Selected imaging clinical findings of this admission are presented in Fig. 1.

The child was transferred to the PICU with a fever peak of 40.5 °C, paroxysmal cough, increased CRP and WBC, then he was given sulfasicillin, vancomycin (day 3 to 9), cefazoxime, meropenem, methylprednisolone sodium succinate (40 mg, QD, from day 2d), phosphocreatine for nutritional support, natriuretic heparin calcium for anticoagulant(0.4 ml, Q12H, day 2 to day 8), and human albumin (5 g, Q12H, day 8 to day 10) for supplemental support, acetylcysteine nebulized inhalation, and sputum chemotherapeutics.

On day 9, he was transferred to Department of Nephrology and Immunology.

**Fig. 2:**
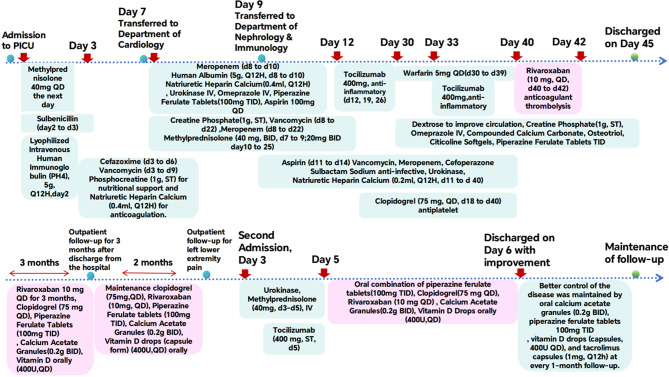
Timeline of the child’s first and second hospitalization medications. Timeline of the child’s medication at first and second admission, with key time points for rivaroxaban administration in red

He was given provisional administration of meropenem and vancomycin for anti-infection, urokinase for thrombolysis, natriuretic heparin calcium for anticoagulation, and sodium creatine phosphate for myocardial protection. On the 15th day of admission, CDFI showed thrombosis with lumen occlusion in the popliteal vein to the common iliac vein of the right lower extremity, and thrombosis with lumen occlusion in the popliteal vein to the common iliac vein of the left lower extremity. Ultrasound showed hyperechoic upper pole of the left kidney alerting for infarction, and enhanced echogenicity of the parenchyma of both kidneys. The bladder wall was slightly rough. Timeline of clinical trajectory, therapeutic interventions, and monitoring outcomes are presented in Fig. 2.

We gave the child the following diagnosis:

(a) sepsis (b) right ventricular thrombus (c) iliac vein embolism (d) pulmonary artery embolism (e)pulmonary hypertension (f) pneumonia (g) pleural effusion. (h) pericardial effusion (i) moderate anemia (j) neutropenia (k) renal vein embolism.

After we confirmed that there was no active bleeding, he was discharged in good basal condition. Rivaroxaban (10 mg, QD, 3 months), clopidogrel, piperazine ferulate, calcium acetate granules and vitamin D were taken orally after this discharge.

**Fig. 3:**
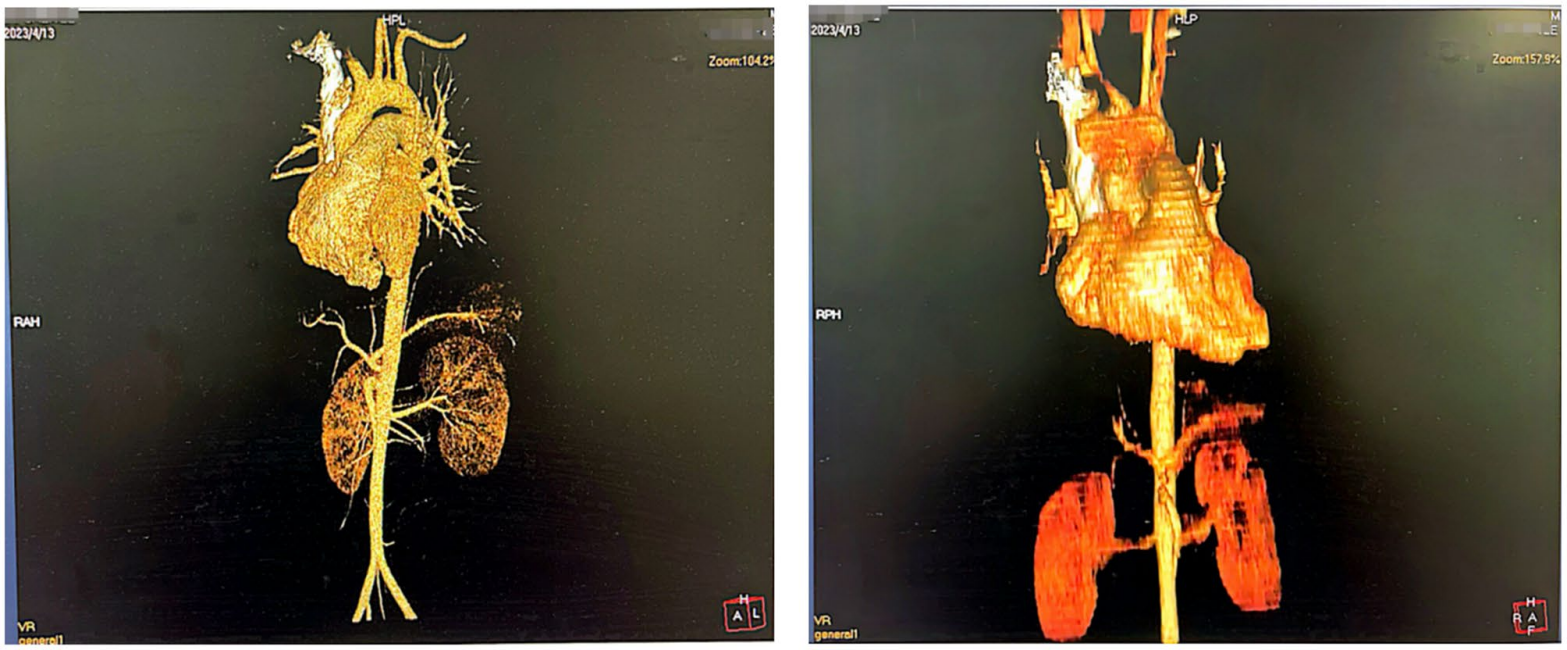
CTA of the chest and abdomen. No effusion shadow was seen in the pleural cavity on both sides. Filling defects of varying sizes were seen in the distal part of the original right pulmonary artery trunk and its branches, and in the left lower pulmonary artery and its branches, which had disappeared. All cardiac cavities were well filled with contrast medium, and a mass-like filling defect was still seen in the right ventricle, with a cross-sectional size of about 7 mm x 5 mm, slightly reduced from the previous one, and the atrial septum was not obviously defective, and the left vertebral artery originated from the aortic arch. The right diaphragmatic surface was slightly elevated

The child’s blood pressure was 112/73 mmHg on readmission review. A grade II/6 systolic murmur was detectable in the 2nd-3rd intercostal space at the left border of the sternum. His leg circumference was 43 cm in both thighs and 33 cm in both calves. Urine and fecal examinations were unremarkable. His coagulation results showed PT 15.9 s, INR 1.29, APTT 50.4 s, and D-dimer 1 160 µg/L. We gave him continued maintenance clopidogrel (once daily), rivaroxaban (once daily), piperazine ferulate tablets (three times daily), calcium acetate granules (twice daily), and vitamin D drops (once daily). CTA findings from this readmission are presented in Fig. 3.

He was admitted to the hospital 9 months later with “intermittent fever for 3 days”, with a temperature of 36.3℃, pulse of 120 beats/min, respiration of 20 beats/min and blood pressure of 107/75 mmHg. The left dorsalis pedis artery was not palpable. The circumference of the left thigh was 50 cm, and that of the right thigh was 51.5 cm. The circumference of both left and right calves was 33 cm.

His blood routine results showed WBC 12.44 × 10^9^/L, ANC 7.44 × 10^9^/L, LY% 17.3%, PLT 587 × 10^9^/L, Hb 127 g/L, CRP 150.77 mg/L, negative ANCA, negative anti-O, and negative MP-antibody. His C3 and C4 were normal. The β2-MG was 0.48 mg/L.

**Fig. 4:**
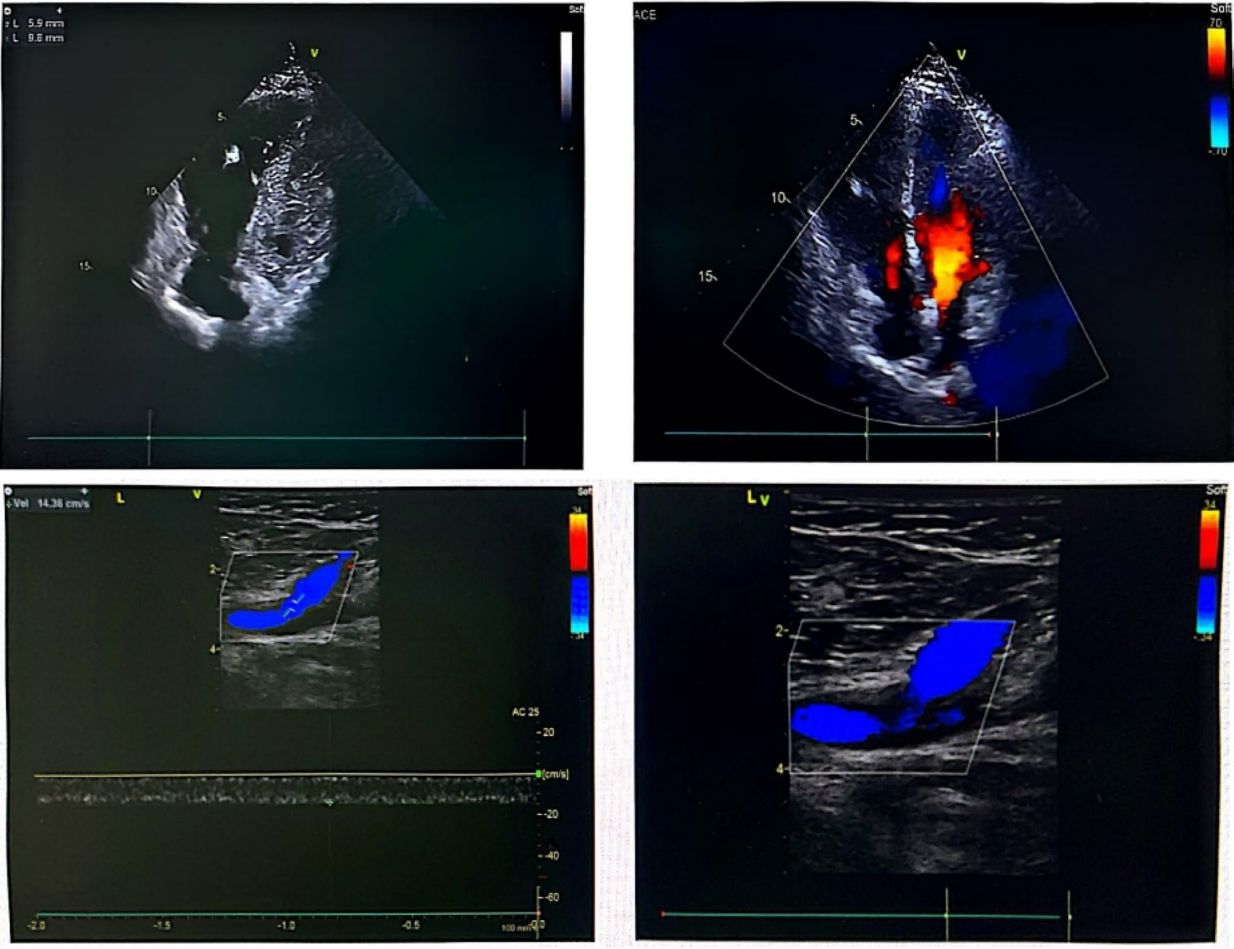
Imaging findings of this admission. UCG showed a 9.8 mm x 5.9 mm hyperechoic mass in the right ventricular cavity, irregular and oscillating with heartbeat. The internal diameters of the right and left pulmonary arteries were 15.3 mm and 13.7 mm, respectively, with an irregular hypoechoic mass proximal to the left pulmonary artery and irregular blood flow distally. CDFI showed thrombosis in the external iliac veins to the common iliac vein of the lower extremities bilaterally, with partial recanalization of the lumen and thrombosis of the distal cardiac appendages. Ultrasound showed thickening of the wall of the bladder, and there was no abnormality of the liver, gallbladder, pancreas, or spleen

Coagulation routines showed PT 15.1 s, APTT 58.9 s, FIB 8.540 g/L, D-dimer 1 930 µg/L, and FDP 5 400 µg/L. New tetralogy of thrombosis showed TAT 1.10ng/mL and PIC 0.45ug/ml. The UCG and CDFI results from this admission are presented in Fig. 4.

**Fig. 5:**
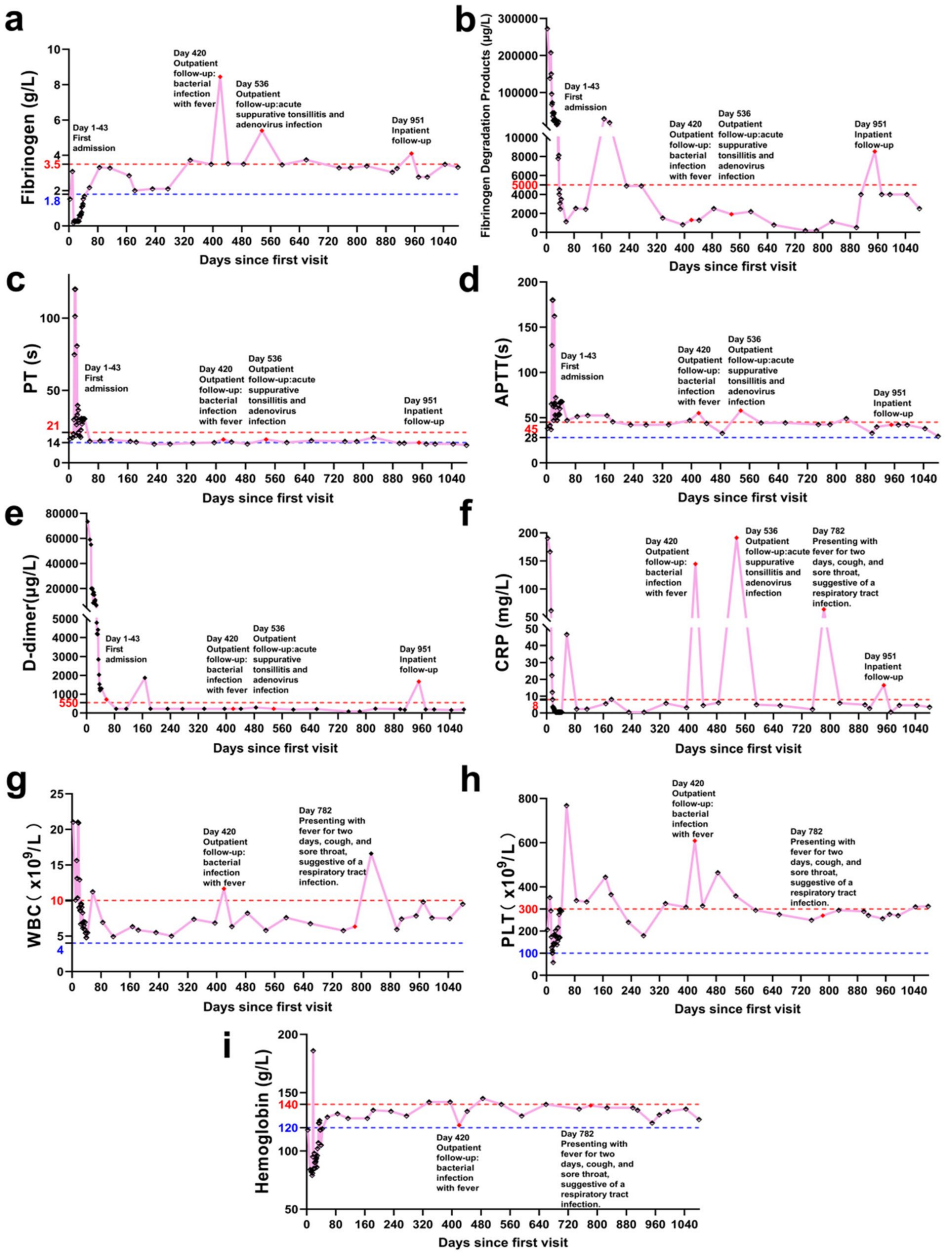
The line chart illustrates the changes in FIB, FIB degradation products, PT, APTT, D-dimer, CRP, WBC, platelet count, and hemoglobin during the follow-up period in children. (**a**) The trend of the child’s Fibrinogen levels from the first follow-up to the present. The blue dashed line indicates the lower limit of the normal range, and the red dashed line indicates the upper limit. (**b**) The trend of the child’s Fibrinogen degradation products from the first follow-up to the present. (**c**) The trend of the child’s PT from the first follow-up to the present. (**d**) The trend of the child’s APTT from the first follow-up to the present. (**e**) The trend of the child’s D-dimer from the first follow-up to the present. (**f**) The trend of the child’s CRP from the first follow-up to the present. (**g**) The trend of the child’s WBC from the first follow-up to the present. (**h**) The trend of the child’s PLT from the first follow-up to the present. (**i**) The trend of the child’s Hemoglobin from the first follow-up to the present

We gave the child sequential dose-reduced intravenous treatment with methylprednisolone, gastroprotection with famotidine, anti-infection with piperacillin, and anticoagulation with urokinase. On day 6 after discontinuation of intravenous methylprednisolone, the sequential dose was reduced to prednisone acetate 10 mg orally three times a day and consolidated with urokinase anticoagulation. Monthly outpatient follow-up of the child was continued. Figure 5 illustrates the trends of routine coagulation parameters and inflammatory markers in our patient over a 1080-day follow-up period.

**Fig. 6:**
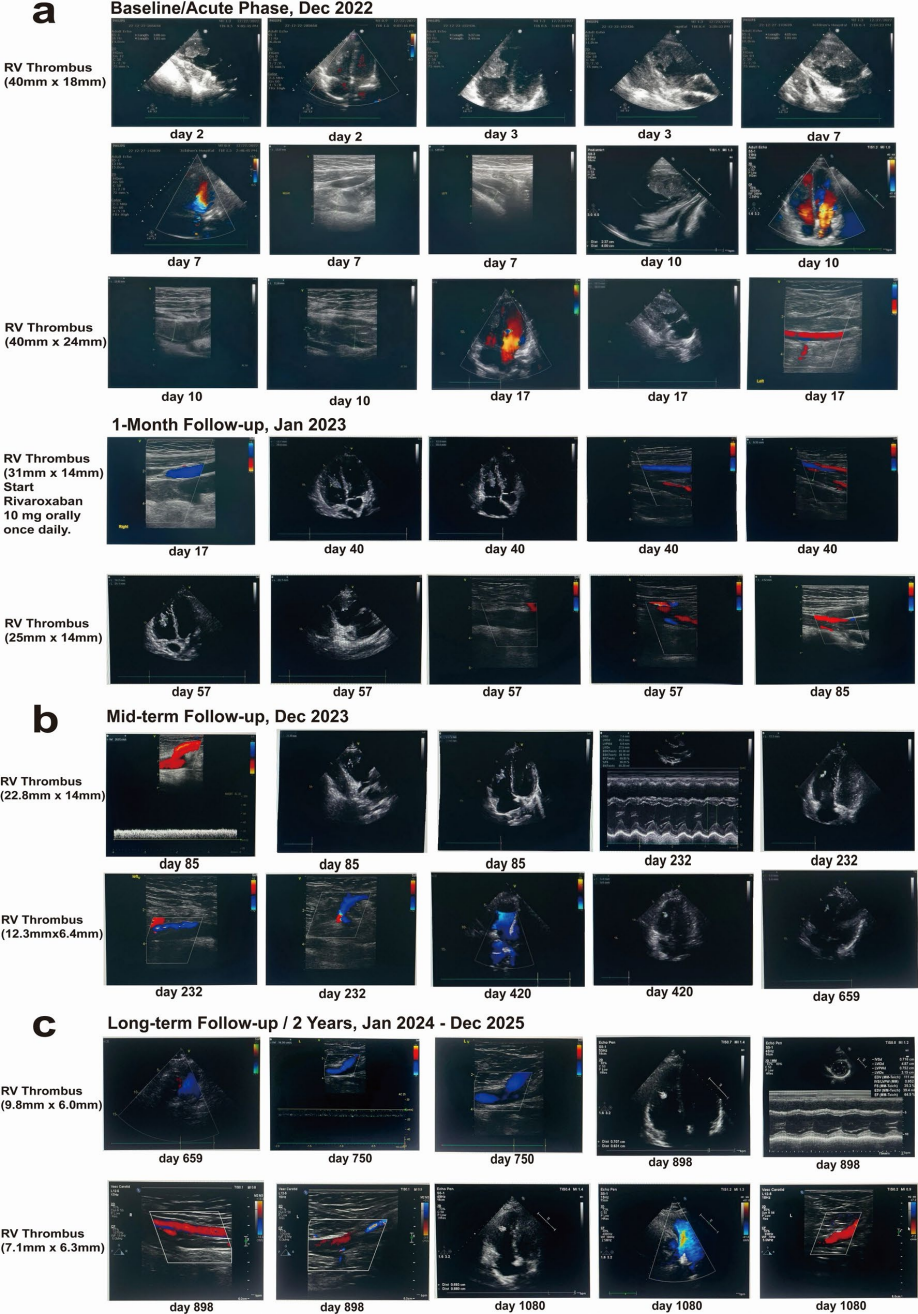
Timeline of echocardiography and CDFI changes throughout disease course and treatment. (**a**) Trends of echocardiogram and CDFI in children admitted for the first time within 0–43 days, with the patient starting rivaroxaban 10 mg once daily on day 40. (**b**) During the middle stage of treatment, subsequent regression of the thrombus. (**c**) During a 2-year follow-up in the long-term treatment process, echocardiography showed regression of thrombus size

His most recent follow-up blood count showed normal WBC, normal RBC, PDW 10.8%, PLT 464 × 10^9^/L, PCT 0.45%, normal CRP, PT 13.1 s, D-dimer 290 µg/L and FDP less than 2 500 µg/L. A hypoechoic mass within the right ventricle measuring approximately 8.9 mm x 5.0 mm was reduced by 77.75% and 72.22% compared to 40 mm × 18 mm at the first time of admission. The timeline of changes in the echocardiography and CDFI findings throughout the disease course and treatment is shown in Fig. 6.

Currently, the child comes to the outpatient clinic for follow-up visit every 2 months. The thrombotic condition has not worsened, and the patient’s overall condition remains good.

The child has been followed up for a total of three years, and is currently maintained on clopidogrel (QD), rivaroxaban tablets (QD), piperazine ferulate tablets (TID), vitamin D drops (QD) and tacrolimus capsules (Q12h), while the hormones and merti-macrolide have been discontinued.

## Monitoring protocol and safety assessment

To ensure the safety and efficacy of the off-label use of rivaroxaban in our patient, a rigorous, multidisciplinary monitoring protocol was implemented throughout the 36-month treatment duration.

### Laboratory monitoring strategy

Acute Phase (Inpatient, Days 0–43): High-frequency monitoring was conducted every 2 days, including Complete Blood Count (CBC) and coagulation profiles (PT, APTT, Fibrinogen, D-dimer). This aimed to evaluate the immediate hemostatic response, screen for heparin-induced thrombocytopenia (HIT) during the bridging phase, and detect early bleeding risks.

Subacute Phase (Months 1–6): Post-discharge monitoring transitioned to a monthly schedule. Tests focused on renal and liver function (ALT, AST, Creatinine) alongside coagulation markers.

Maintenance Phase (Months 7–36): Once the patient achieved clinical stability and D-dimer levels normalized (< 500 µg/L), the monitoring interval was extended to every 2 months for long-term safety surveillance.

### Imaging surveillance protocol

Modalities: Transthoracic Echocardiography (TTE) was utilized to quantify the dimensions and morphology of the right ventricular thrombus. Simultaneously, Doppler Ultrasound was employed to assess the recanalization status of the bilateral iliac, femoral, and popliteal veins.

Frequency: Imaging was performed at baseline, followed by monthly reviews during the first 6 months to track early thrombus regression. From 12 to 36 months, comprehensive imaging assessments were conducted every 1.5–2 months. This schedule allowed for dynamic adjustment of the treatment regimen based on the rate of thrombus resolution.

### Clinical safety assessment

At every outpatient visit, a structured checklist was used to screen for signs of overt or minor bleeding (e.g., epistaxis, gingival bleeding, ecchymosis, hematuria). Medication adherence and potential drug-drug interactions were also rigorously evaluated at each encounter to ensure optimal therapeutic outcomes.

ASH issued guidelines in 2024 recommending the prioritized use of DOACs (such as rivaroxaban) in eligible pediatric patients and emphasizing ongoing assessment of treatment response. The monitoring in our overall protocol is more stringent than the routine recommendations in the guidelines [1]. 

## Discussion and narrative review

Venous thromboembolism (VTE) refers to disorders of impaired venous return in which blood clots abnormally in the veins, resulting in complete or incomplete obstruction of the vessel, including deep vein thrombosis (DVT) and pulmonary embolism (PE). The annual incidence of VTE in pediatric patients is approximately 0.14 to 0.21 per 10 000, but the rate increases to 0.2 to 0.6% in hospitalized children, with pulmonary embolism accounting for approximately 15% of these cases [2]. According to the 2018 U.S. National Trauma Data Bank, the incidence of VTE in children increases with age——0.1% for those under 12 years old, 0.3% for those 13–15 years old, and 0.8% for those 16 years old and older [3]. 

**Fig. 7:**
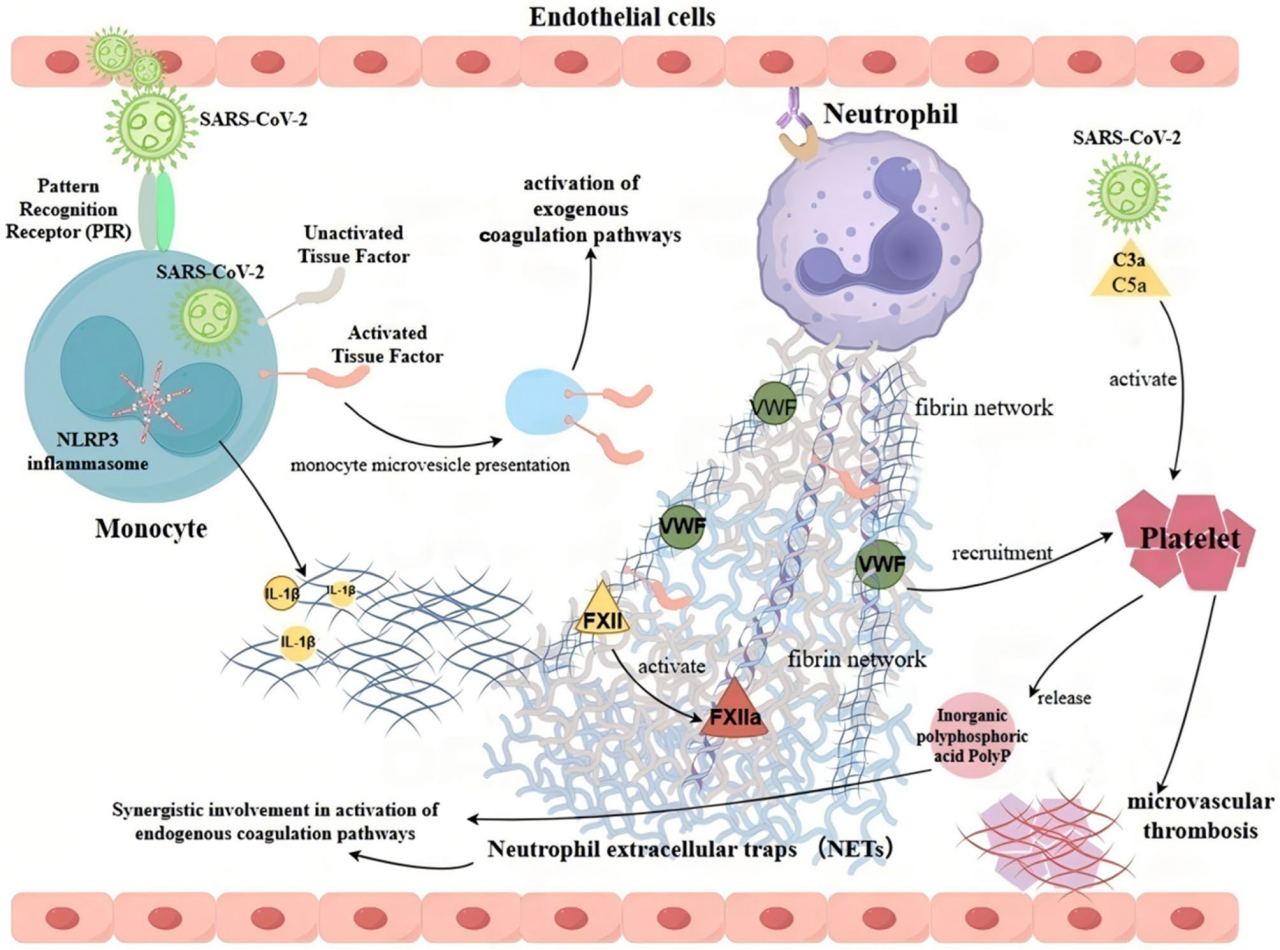
SARS-CoV-2 activates the body’s intrinsic immune response, leading to unrestricted thrombin generation. SARS-CoV-2 infection of endothelial cells induces cellular pyroptosis, platelet-vessel wall interactions that increase endothelial damage, release of inorganic polyphosphate from activated platelets and neutrophils, and an extracellular bactericidal network of neutrophils, which ultimately lead to unrestricted thrombin generation and thrombus formation. Platelet-vessel wall interactions mediated by surface receptors (integrins and selectins) and adhesion proteins (von Willebrand Factor and fibrinogen) compromise vessel wall integrity or increase endothelial damage. Activated immune cells release reactive oxygen species and pro-inflammatory mediators that further impair endothelial function and exacerbate microvascular dysfunction [11, 12]

Previous studies by Bray et al. suggested that COVID-19 increased the risk of thrombotic complications in patients by inducing immunothrombosis [4–7]. Coronavirus infection of endothelial cells induces cellular pyroptosis, leading to the release of pathogen-associated molecular patterns (PAMPs) and damage-associated molecular patterns (DAMPs), and a dysregulated immune response that further exacerbates microvascular dysfunction. IL-6 and TNF-α upregulate the expression of adhesion molecules and tissue factor on endothelial cells, promoting activation of the coagulation cascade and thrombosis in the microvasculature [8, 9]. Aiping et al. found that while NLR and IL-6 levels are indeed important biomarkers for predicting disease severity and survival, the duration of illness in patients with severe COVID-19 is positively correlated with serum IL-8 and soluble IL-2Rα levels [10]. IL-8 is a key neutrophil chemotactic factor, and its association suggests that neutrophils continue to play a role in the progression of the disease—possibly by recruiting neutrophils or through PMN-MDSC-mediated suppression of T-cell responses [10]. Proposed pathophysiological mechanism of SARS-CoV-2 associated immunothrombosis is depicted in Fig. 7.

Our patient’s specific laboratory profile provides evidence linking the SARS-CoV-2 infection to the thrombotic event via immunothrombosis. While general markers like CRP were elevated, the specific elevation of IL-8 (86.8 pg/mL) is particularly revealing. IL-8 is a key chemotactic factor for neutrophils. As described by Ma et al. and Chang et al., elevated IL-8 in severe COVID-19 correlates with neutrophil overactivation and the release of NETs [10, 13]. These NETs act as a scaffold for thrombosis, trapping platelets and fibrin, which directly explains the extensive multi-system thrombi observed in our patient despite the absence of prior thrombophilia. Furthermore, the initial hypofibrinogenemia (1.52 g/L) observed on admission suggests a consumptive coagulopathy driven by this intense thrombo-inflammatory process, rather than simple stasis-induced thrombosis.

The age of onset of DVT in children has a bimodal distribution, and the risk factors for its formation include: age less than 1 year and greater than 13 years, central venous cannulation, cachexia, trauma, and burn infection [14, 15]. Alan S Go et al. showed a nearly 3.5-fold increased risk of SARS-CoV-2-associated VTE in a multivariate analysis of hospitalized patients; the cumulative incidence of VTE in critically ill patients with pulmonary embolism complications was approximately 25%-49%.VTE may be a major cause of death in critically ill patients in the post-epidemic era [16]. In addition, acute myocardial injury, pulmonary microvascular thrombosis and arterial thromboembolism were common complications in patients.

Plasma D-dimer is being used as a prognostic marker for post-COVID VTE administration, and D-dimer above 1000 µg/L is an independent risk factor for in-hospital mortality in patients [17]. Current evidence suggests that a positive D-dimer result after three months of anticoagulant therapy in patients experiencing their first unprovoked VTE is associated with a twofold increased risk of VTE recurrence compared to a negative result [18]. Conversely, persistently elevated D-dimer levels following the cessation of a three-month anticoagulant regimen support the continuation of therapy in cases of unprovoked VTE [19]. The plasma levels of post-infection hypercoagulable markers, such as D-dimer and thrombin-antithrombin complex, represented by SARS-CoV-2, are significantly elevated. This simultaneously reflects infection-related endothelial dysfunction (such as modulating vascular integrity, remodeling extracellular matrix) and the inflammatory state, which drives patients towards a hypercoagulable state, translating into an increased risk of thromboembolism and cardiovascular events [20, 21]. 

Based on the clinical trajectory, we retrospectively confirmed the diagnosis of Multisystem Inflammatory Syndrome in Children (MIS-C). The patient met the rigid criteria defined by the CDC and WHO [22–24], presenting with: persistent fever > 38.0℃; severe systemic inflammation (CRP 222.72 mg/L, Ferritin 717 pmol/L, IL-1 RA 2683 pg/ml (0-2171 pg/ml).

IL-8 86.8 pg/ml (0–47 pg/ml)); multi-organ involvement including cardiac (RV thrombus), renal (renal vein thrombosis), hematologic (coagulopathy), gastrointestinal systems (abdominal pain) and evidence of recent SARS-CoV-2 infection. Distinct from isolated COVID-19 hypercoagulability seen in adults——which is often limited to pulmonary vasculature, this patient exhibited a systemic “cytokine storm” phenotype with profound myocardial and renal involvement. Furthermore, sepsis-associated coagulopathy was ruled out due to negative serial blood cultures and the specific responsiveness to immunomodulatory therapy such as IVIG and corticosteroids rather than antibiotics alone. The recognition of MIS-C is pivotal, as it dictates the need for aggressive dual therapy consisting of anticoagulation plus immunomodulation to resolve the immunothrombosis.

Long-term complications of venous thromboembolism in children include post thrombotic syndrome (PTS), loss of central venous access, and chronic thromboembolic pulmonary hypertension [25]. 

Direct oral anticoagulants (DOACs) such as apixaban, rivaroxaban, edoxaban, and dabigatran are commonly used to treat patients with venous thromboembolism [26]. 

The EINSTEIN-Jr Phase III study was the first oral anticoagulant trial in children and the largest study of anticoagulation therapy in children. While the adult VTE trial exclusively focused on symptomatic proximal deep vein thrombosis (DVT) and pulmonary embolism (PE), the JUNIOR cohort encompassed a broader spectrum of thrombotic phenotypes. These included cerebral venous thrombosis (CVT), catheter-associated VTE, and thrombi localized in atypical sites such as the renal vein, portal vein, right cardiac chambers, and jugular vein. Additionally, the study population featured both catheter-related and non-catheter-related VTE, with interventions ranging from initial heparinization to thrombolysis or thrombectomy, reflecting the heterogeneity of pediatric thrombosis presentations [27]. Children in group of rivaroxaban received body mass-adjusted equivalents of the total daily dose of 20 mg in adults with 70 kg body mass (1 time daily for body mass ≥ 30 kg, 2 times daily for body mass 12 kg ≤ 30 kg, and 3 times daily for body mass < 12 kg), and children in the standard anticoagulant group were continued on heparin or switched to a VKA. The results demonstrated that body mass-adjusted rivaroxaban for the treatment of venous thromboembolism in children had a low risk of recurrence, reduced thrombotic burden, and no increased risk of bleeding. It avoided the use of adult dosage forms and significantly reduced the number of injections required for standard anticoagulation therapy and associated blood sampling [27]. 

Safety data on rivaroxaban in pediatrics have been accumulating gradually [27–32]. Hassan and Motwani’s study showed an overall recurrence rate of 3.6% in 52 children aged 0 to 16 years treated with rivaroxaban for VTE, with no bleeding events [33]. Natalie Montanez et al. describe the anticoagulation management of an 8-year-old boy with right-sided dilated coronary artery aneurysms secondary to Kawasaki disease who remained stable on rivaroxaban and aspirin therapy after bleeding complications from enoxaparin and warfarin provocation. The use of rivaroxaban appears to be safe and effective in preventing thrombosis in pediatric patients with CAA [34]. McElroy et al. showed that the incidence of bleeding in rivaroxaban-treated children was 11.8%, which is similar to that of low-molecular-weight heparin (LMWH) at 9.3% but significantly lower than that of the direct thrombin inhibitors (DTIs) at 50%, of heparin at 38.1%, and of warfarin at 25% [35]. Recurrence of venous thrombosis occurred in 1% of children with VTE treated with rivaroxaban, compared with 3% treated with standard anticoagulants [27]. The assessment of absolute and relative efficacy and safety of rivaroxaban compared with standard anticoagulants was similar to the results in the adult rivaroxaban study [36–38]. Reviewing this child, there was no thrombotic recurrence at the 3-year follow-up, and D-dimer decreased from 73 430 µg/L to 290 µg/L, further supporting its potential long-term effectiveness.

Current pediatric anticoagulation guidelines are largely based on low-quality evidence [39, 40]. Adult guidelines recommend rivaroxaban as the first-line treatment for VTE [19], such as 2023 ISTH guideline suggests that post-discharge thromboprophylaxis with a prophylactic dose of rivaroxaban may be considered for approximately 30 days to reduce the risk of VTE following hospitalization for COVID-19, especially for patients with persistent VTE risk factors, such as a high IMPROVE risk score or elevated D-dimer levels [41]. However, pediatric recommendations still rely on single-arm studies (e.g., EINSTEIN-Jr) and expert consensus [35, 39, 42]. A Guideline for the Pharmacologic Treatment of Thrombophilia in Children, which uses the Delphi method to reach consensus on recommendations and level of evidence, states that anticoagulants are recommended for children with symptomatic DVT or PTE. The initial therapy (first 5–10 days) is about initial acute phase therapy in children with newly diagnosed VTE, parenteral gastrointestinal anticoagulation with LMWH for at least 5 days is recommended. Follow-up (after the first 5–10 days) for children ≥ 12 years old, maintenance therapy with DOAC is recommended(e.g., rivaroxaban, dabigatran). For patients between 2 and 12 years old, DOAC or LMWH is recommended(well-established, evidence-based). For patients < 2 years old, LMWH is recommended (strong recommendation, moderate-quality evidence) [43]. In addition, there is a paucity of data on the interaction of rivaroxaban with immunosuppressive agents (e.g. tocilizumab), and one needs to be wary of fluctuations in efficacy due to hepatic enzyme induction [44–46]. Sidra A Hasan et al. reported a case of improvement in prognosis of acute limb ischemia (ALI) in a patient using TCZ and heparin. Cytokine release syndrome (CRS) was treated with TCZ. They administered TCZ (6 mg/kg) intravenously from the first day of hospitalization in response to CRS. They found a significant decrease in CRP levels on day 2 after TCZ treatment [47]. Current evidence on direct oral anticoagulants for pediatric VTE is largely derived from the EINSTEIN-Jr trial which validated rivaroxaban’s non-inferiority to standard anticoagulants [28]. While the EINSTEIN-Jr trial suggests a weight-adjusted dose of 15 mg for children weighing 30–50 kg, we opted for a reduced dose of 10 mg based on a personalized risk-benefit assessment. Specifically, during the acute phase, the patient presented with significantly prolonged APTT, indicating a heightened risk of hemorrhage. Although standard protocols for VTE treatment typically recommend 15–20 mg/day for adults [48], and pediatric equivalents are similar, we prioritized safety by de-escalating the dose. This approach aligns with the ISTH Guidelines for Antithrombotic Treatment in COVID-19, which advocate for vigilant bleeding risk assessment and individualized dosing in COVID-19-associated coagulopathy——post-discharge rivaroxaban (10 mg daily) may be considered for patients with persistent VTE risk factors (IMPROVE score ≥ 4 or 2–3 with elevated D-dimer) and no contraindications [49]. There were no bleeding or thrombotic exacerbation events with the combination of rivaroxaban and tocilizumab in our case, suggesting that 10 mg regimen provided a safe therapeutic window for this specific MIS-C presentation, but a larger sample size is needed for validation.

Our report aligns with emerging reports of rivaroxaban efficacy in children yet diverges in aspects. Firstly, the initial thrombus volume (40 × 18 mm in the right ventricle) exceeds reported pediatric cases (median 12 × 8 mm) [50]. Secondly, this follow-up provides critical safety data, with D-dimer reduction from 73 430 µg/L to 290 µg/L and no bleeding complications—a finding consistent with adult studies but rarely documented in children. Furthermore, unlike localized DVT in prior reports, the child involved four vascular territories, mirroring findings of diffuse microthrombosis in fatal COVID-19. Despite consensus guidelines recommending DOACs for children ≥ 12 years [43], their use in younger patients and complex cases remains off-label. Our findings support the Delphi-based pediatric thrombophilia guidelines [51], demonstrating rivaroxaban’s feasibility in high-risk settings. Current weight-based protocols may underestimate the requirements for hypercoagulable states. While the volume of data cases is sparse, only 23% of pediatric VTE studies report ≥ 1-year follow-up [50, 52–54]. Additionally, no pediatric guidelines address anticoagulation duration in post-COVID VTE. The 2025 American Society of Hematology guidelines for COVID-19 thromboprophylaxis prioritize five key research areas. These include conducting high-quality multicenter randomized trials and investigating how non-anticoagulant interventions such as vaccines and corticosteroids influence thrombotic risk. Additionally, research should examine the impact of viral variants, validate risk assessment models for thrombosis and bleeding, and further evaluate the clinical outcomes of anticoagulation therapy [55]. 

Routine screening for coagulation markers is essential for coronavirus infections, consecutive relapses and remissions lasting at least 3 months after infection, or for progressive disease states (first-time blood count and significant elevation of CRP or other inflammatory markers). Additional studies and follow-up data collection are needed for thrombotic events and the clinical use of DOACs in children to explore long-term treatment options for thrombophilia in children. While rivaroxaban offers a promising therapeutic avenue, its application must be guided by rigorous monitoring and shared decision-making with families.

This study has several limitations. First, as a single-center retrospective case report, the findings regarding the efficacy of rivaroxaban are anecdotal and cannot be generalized without larger cohort studies. Second, while genetic testing for thrombophilia was negative, potential unknown genetic predispositions cannot be entirely ruled out. Finally, the optimal duration of anticoagulation for post-COVID-19 MIS-C associated thrombosis remains undefined, and our 3-year strategy was based on clinical judgment rather than established guidelines. As the medical community navigates the long-term sequelae of the pandemic, studies like this support the imperative to bridge evidence gaps in pediatric rare diseases—ensuring equitable care for all children, regardless of disease rarity. 

## Data Availability

The data that support the findings of this study are available on request from the corresponding author. The data are not publicly available due to privacy or ethical restrictions.

## Abbreviations

ALT: Alanine Aminotransferase
ANC: Absolute Neutrophil Value
APTT: Activated Partial Thromboplastin Time
AT-III: Antithrombin III
C3: Complement 3
C4: Complement 4
CDFI: Color Doppler Examination
CRP: C-reactive Protein
CTA: Computed Tomography Angiography
DBIL: Direct Bilirubin
FDP: Fibrin/Fibrinogen Degradation Products
FIB: Fibrinogen
Hb: Hemoglobin
Hs-cTn: Ultra-sensitive Troponin T
IgA: Immunoglobulin A
IgG: Immunoglobulin G
INR: International Normalized Ratio
KET: Urine Ketone Bodies
LY: Lymphocyte
LY%: Lymphocytes Percentage
MPV: Mean Platelet Volume
MYO: Myoglobin
N: Neutrophil
Neutrophil Extracellular Traps: NETs
NEUT%: Neutrophil Percentage
PCT: Thrombocytocrit
PDW: Platelet Distribution Width
PIC: Fibrinolysis-alpha2 Fibrinolytic Inhibitor Complexes
PLT: Blood Platelet
PT: Prothrombin time
PTR: Partial Thromboplastin Time Ratio
SAA: Serum Amyloid A
TAT: Thrombin-antithrombin Complex
TBIL: Total Bilirubin
TM: Thrombomodulin
TT: Thrombin Time
UCG: Ultrasonic Cardiogram
WBC: White Blood Cell
β2—MG: β2-microglobulin
